# Cardiovascular Conditions of Patients on HIV Therapy

**DOI:** 10.36660/abc.20190810

**Published:** 2020-01

**Authors:** Alfredo José Mansur

**Affiliations:** Instituto do Coração - Faculdade de Medicina da Universidade de São Paulo - Cardiologia Clínica - Ambulatório Geral, São Paulo, SP - Brazil

**Keywords:** HIV, HIV Infections, Cardiovascular Diseases, Antiretroviral Therapy, Risk Factors, Carotid Artery Diseases, Atherosclerosis, Epidemiology

Successful anti-infective therapy of patients submitted to contemporary HIV treatment decreased mortality between 2007 and 2017 by 51% in association with a decrease in of 17% in incidence; decrease in mortality associated with a smaller decrease in incidence means more people living with HIV disease.^1^ In Brazil, annualised rate of change in mortality was -1.2% (-1.4% to -1.0%).^2^ In many countries the survival rate of patients increased.^3^ Therefore, other non-infectious ailments such as prevalent cardiovascular diseases came to the attention of physicians in charge for the patients.^4^

Prevention is a mainstay in the health care of patients. Patients on successful HIV treatment may incur in either asymptomatic or symptomatic conditions that may be risk factors for cardiovascular diseases^5^ or demonstrate metabolic abnormalities^6^ that need medical attention. Further, new technologies were studied in order to investigate in more depth vascular health either as screening, or a diagnostic tool or treatment strategies.^3^

Previous research were performed in Brazilian populations of different geographic regions. Median carotid intima-media thickness was 0.54 (0.49, 0.62) mm in 535 HIV infected patients from Rio de Janeiro, 0.58 (0.52, 0.68) mm in 88 healthy controls and 0.57 (0.49, 0.70) mm in 10943 participants of a large cohort; differences were not significant after adjustment for confounding variables.^7^

In Parana state, in a sample of 538 patients, hypertension was diagnosed in 24.4%, hypercholesterolemia in 18.2%, low HDL-cholesterol in 39.7%, hypertriglyceridemia in 51.3% and high serum glucose in 33.3%.^5^ In Minas Gerais state a study of a cross-sectional sample of 133 patients compared with 20 healthy controls demonstrated that insulin resistance was more common among the infected patients, and suggested lipid accumulation product index as a new biomarker of cardiovascular risk in patients with HIV.^8^

In the current issue of *Arquivos Brasileiros de Cardiologia*, Leite et al.^9^ report an additional Brazilian experience in Recife, Pernambuco State. They evaluated a convenience sample of 99 asymptomatic patients with low cardiovascular risk and undetectable plasma HIV RNA levels on HIV treatment in comparison to 16 controls for a set of inflammatory biomarkers - IFN-g, TNF-a, IL-1b, IL-6, sVCAM-1 e sICAM-1 and carotid intima-media thickness. After multivariate analysis, they found a significant association between TNF-a and IL-1b with the risk for higher carotid-intima media thickness in HIV infected patients. They reproduced the negative findings of no difference relative to carotid intima-media thickness between the study groups. These findings add to the evaluation of patients on successful HIV therapy and re-emphasizes the achievements of the contemporary comprehensive clinical care of the patients probably including the therapeutic advice of cardiovascular risk factors such as smoking, hypertension, dyslipidemia and diabetes.
